# Measurement of vitreous humor pressure in vivo using an optic fiber pressure sensor

**DOI:** 10.1038/s41598-023-45616-z

**Published:** 2023-10-25

**Authors:** Masashi Mimura, Tadamichi Akagi, Ryosuke Kohmoto, Yasushi Fujita, Yohei Sato, Tsunehiko Ikeda

**Affiliations:** 1 Department of Ophthalmology, Osaka Medical College, Takatsuki-City, Osaka Japan; 2 Department of Ophthalmology, Hyogo Medical University, 1-1, Mukogawa-Cho, Nishinomiya-Shi, Hyogo 663-8501 Japan; 3 Department of Ophthalmology, Toho University Sakura Medical Center, Sakura-City, Chiba, Japan; 4 Division of Ophthalmology and Visual Science, Niigata University Graduate School of Medical and Dental Sciences, Niigata, Japan

**Keywords:** Ocular hypertension, Preclinical research

## Abstract

We conducted a study to assess the pressure difference between the aqueous and vitreous humors in rabbit eyes using a direct intraocular pressure (IOP) measurement method. A micro-optic-fiber pressure sensor was utilized for this purpose. Preliminary experiments with enucleated porcine eyes confirmed the sensor's accuracy in measuring both aqueous and vitreous humor pressure. The main study involved six healthy albino rabbits, where the sensor measured the pressure in the anterior chamber (aIOP) and posterior vitreous-cavity (pIOP). These measurements were compared to aIOP values obtained through rebound tonometry. Additionally, pre- and postoperative pressure comparisons were made after performing a vitrectomy. Results revealed a significant disparity between aqueous and vitreous humor pressures. Prior to vitrectomy, pIOP was 22.8 mmHg, over twice as high as aIOP (11.0 mmHg), but decreased to a similar level following the procedure. Comparison between the sensor measurements and rebound tonometry showed agreement in aIOP values. In conclusion, our study demonstrates that vitreous humor pressure is consistently higher than aqueous humor pressure, reaching the upper limit of normal IOP. Furthermore, vitrectomy effectively reduces pIOP, aligning it with aIOP. These findings contribute valuable insights into intraocular pressure dynamics and have implications for clinical interventions targeting ocular pressure regulation.

## Introduction

Intraocular pressure (IOP) is one of the most fundamental parameters of eye pathophysiology and has been measured by transcorneal tonometry by using applanation, indentation, rebound, and pascal dynamic contour tonometer^1^. The assessment of IOP using the tonometer focuses on measuring the anterior chamber pressure (aIOP) via corneal distortion by external compression; however, the posterior vitreous-cavity pressure (pIOP) has not yet been investigated in detail. From the standpoint of aqueous humor circulation, both chambers are connected such that the flow is provided on the basis of the production–drainage balance by an equivalent rate known as the Goldmann equation (IOP = [aqueous humor formation/outflow] + episcleral venous pressure)^2–4^ so that the aqueous humor pressure in both cavities is anticipated to correspond; this may be one of the reasons to omit the discussion regarding pIOP.

However, the posterior vitreous cavity is mostly filled with gelatinous vitreous, and pIOP can be provided by both vitreous and aqueous humor. Previous studies have demonstrated the analyses of aqueous humor flow of both chambers by using computational fluid dynamics modeling and have shown that a fraction of the aqueous humor secreted from the ciliary body stays in the posterior chambers^5–10^. Therefore, the vitreous humor pressure seems to be essential for regulating pIOP. Addtionally, some reports using the computational modeling have advocated that there is a pressure gradient between the anterior and posterior chamber, thus suggesting that there might be a discrepancy between aqueous and vitreous humor pressure^9,11–13^.

Notably, ex vivo animal studies focused on measuring aIOP and pIOP have been reported recently. Hernandez-Verdejo et al. measured the continuous pIOP of the enucleated porcine eye by using blood pressure transducers attached to 21-gauge catheters during the injection of water into the anterior chamber to increase aIOP from 0 to 180 mmHg, and they found that the pressure was not linear between the two chambers^14^. Gopesh et al. used their original high-resolution pressure sensor attached to a 30 or 31-gauge needle to measure the aIOP and pIOP of rabbit eyes ex vivo^15^; however, the authors from both reports concluded that the sensors did not work well within the vitreous body because of the viscosity of the vitreous humor. Thus, pIOP provided by vitreous humor in vivo remains unknown.

The current study focuses on directly measuring the in vivo pIOP provided by vitreous humor. To this end, we utilized a commercially available micro pressure sensor for medical experiments related to cardiology, craniology, otology, and ophthalmology^16–20^. We aimed to reveal pIOP with and without vitreous by using the sensor and to compare it with an aIOP measured using both the pressure sensor and conventional rebound tonometer to assess whether pIOP can be an important eye-conditioning parameter and to further the understanding of the fundamental dynamics of the eye.

## Material and methods

We utilized a micro fiber-optic pressure sensor (diameter: 0.3 mm) attached to a signal conditioning system (FOP-LS-PT9 and FPI-LS-10 Module on EVO-SD-5 Evolution Chassis, FISO Technologies, Inc., Quebec, Canada), for direct IOP measuring. The system is connected to a computer with an exclusive data logger software (Evolution Software, FISO Technologies, Inc., Quebec, Canada) installed for real-time monitoring and data calculation.

The animals involved in the observational study were handled in accordance with the Association for Research in Vision and Ophthalmology Resolution on the Use of Animals in Research and Animal Research: Reporting of In Vivo Experiments (ARRIVE) guideline. The experimental protocol was approved by the Committee of Animal Use and Care of Osaka Medical College, Japan.

### Preliminary experiment to clarify the properties of the fiber-optic pressure sensor

The optic pressure sensor has some silicone gel (the polymer) that deforms and transduces the pressure to the sensor silicon (the semiconductor) flexible membrane. The signal from the membrane is converted via optical fiber sensing with a Fabry–Perot cavity and is then sent to the main system^21^. To measure the IOP without the leakage of intraocular fluid, a 25-gauge trocar–cannula with a closure valve for microincisional vitrectomy was inserted via limbus for measuring aIOP or pars plana for measuring pIOP to introduce the sensor into the anterior or posterior cavity. The pressure sensor was calibrated before each experiment, following the instruction manual^22,23^. The sensor was inserted 5 mm/10 mm from the tip for measuring aIOP/pIOP to measure the pressure at the center of the anterior chamber/the core of the vitreous body. After confirming that the pressure stabilized on the real-time monitor, we commenced data acquisition (200 times/s for 5 s) to calculate the mean pressure.

To ensure that the measurement using the sensor membrane was accurate both in fluid and vitreous gel, we performed a preliminary experiment. We prepared three enucleated porcine eyes and removed its lens including the posterior capsule and anterior vitreous membrane, to make the eye in one chamber. Then, simultaneous measurement of the anterior chamber and posterior cavity using the fiber-optic pressure sensor was performed under IOP control through an infusion cannula inserted in the anterior chamber. The infusion cannula was connected to a vitrectomy system (Alcon Constellation Vision System), and IOP control was set at 16 mmHg and 33 mmHg, to put the eye in one chamber with consistent normal and abnormal pressure in both aqueous and vitreous humor.

### Main study for measuring aIOP and pIOP using a fiber-optic pressure sensor

We measured the aIOP and pIOP of the 12 eyes of 6 albino rabbits weighing 2.7–3.5 kg (Slc: JW/CSK, Japan SLC Co., Hamamatsu, Japan). To compare the pressure between conventional transcorneal tonometry and the fiber-optic pressure sensor, we also measured aIOP three times by using i-Care TONOVET (ICARE Finland Oy, Vantaa, Finland) in advance. After anesthetizing the rabbits with an intramuscular injection of medetomidine 0.5 mg/kg, midazolam 2 mg/kg, and butorphanol 0.5 mg/kg, a stereotactic head holder was used to stabilize the heads. We then measured aIOP by using a calibrated i-Care (three times each to obtain an average value).

**Figure 1:**
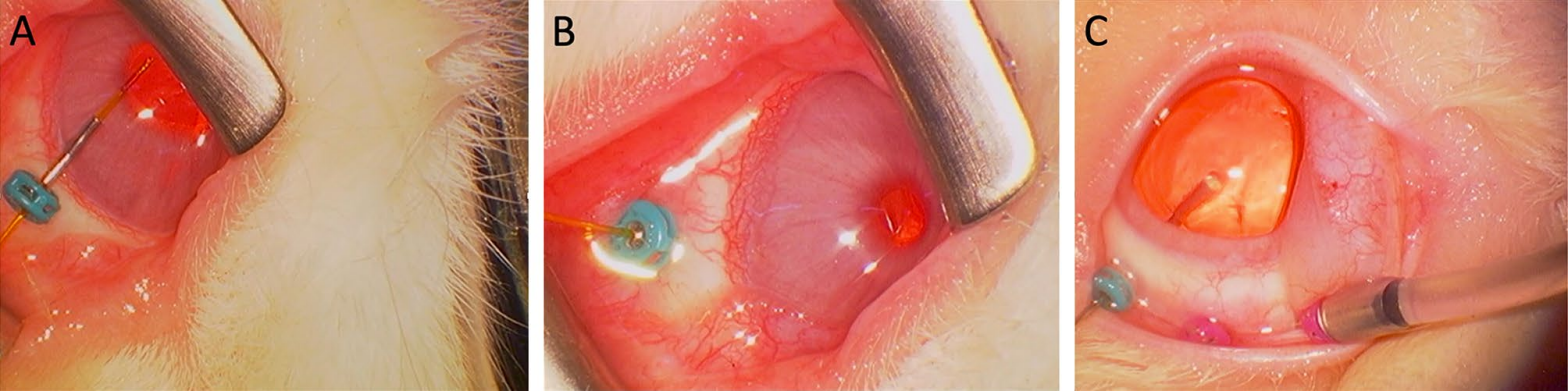
Series of photographs during the measurement using a 25-gauge trocar–cannula with a closure valve for microincisional vitrectomy. (**A**) Introducing a fiber-optic sensor into the anterior chamber via limbus, (**B**) introducing it into the posterior vitreous cavity via pars plana, and (**C**) performing a two-port vitrectomy to remove the core vitreous and anterior vitreous membrane with a vitreous cutter (right cannula) under the irrigation of a balanced salt solution (left cannula).

Subsequently, we measured the aIOP of the eye by using a fiber-optic pressure sensor. As described above, the measurement sensor was inserted into the anterior chamber by using a 25-gauge trocar–cannula with a closure valve via the limbus after the instillation of topical anesthesia with oxybuprocaine hydrochloride (Fig. 1A). After the procedure, the animals were housed in the institutional animal care facility for one week to recover the aIOP, which may be lowered due to the leakeage of the aqueos humor when removing the trocar-cannula^24^. Thereafter, we measured the pIOP by using the same method via the pars plana (Fig. 1B).

### Measurement of aIOP and pIOP after core vitrectomy

After the measurement of the pIOP, we performed a two-port vitrectomy via the 25-gauge trocar cannulas by using a vitreous cutter driven by CV-24000 (NIDEK, Co., Ltd., Aichi, Japan) (Fig. 1C). The surgery was followed by mydriasis by using topical 0.4% tropicamide (Mydrin M; Santen, Osaka, Japan) to remove the core vitreous body, including the anterior vitreous membrane. After the surgery, the animals were housed in the institutional animal care facility again for a week for aIOP recovery. Thereafter, we measured the aIOP and pIOP using the i-Care and pressure sensor.

### Statistical analysis

Statistical analyses were performed using JMP statistical software (version 15.1.0; SAS Institute, Cary, NC). The pressure data are expressed as mean (standard deviation). The Wilcoxon signed-rank test was used to compare the mean data. Differences were considered statistically significant at *P* < 0.05.

## Results

Regarding the preliminary experiment to determine the properties of the optic-fiber sensor, the mean aIOP (aqueous humor) and pIOP (vitreous humor) for both low and high (16 mmHg and 33 mmHg) IOP setting were 16.33 (0.87)/32.29 (1.85) mmHg and 17.35 (0.50)/34.47 (1.12)mmHg, respectively. The two measurements showed no statistical difference in both IOP setting (low and high; *P* = 0.10 and 0.11, respectively). However, the measurement of vitreous gel pressure tends to be slightly higher than the aqueous humor pressure. Thus, as a supplemental experiment, we have prepared one more eye following lensectomy, and changes in aIOP and pIOP were tracked over 40 min. The results revealed a decrease in the difference between aIOP and pIOP over time.

**Figure 2:**
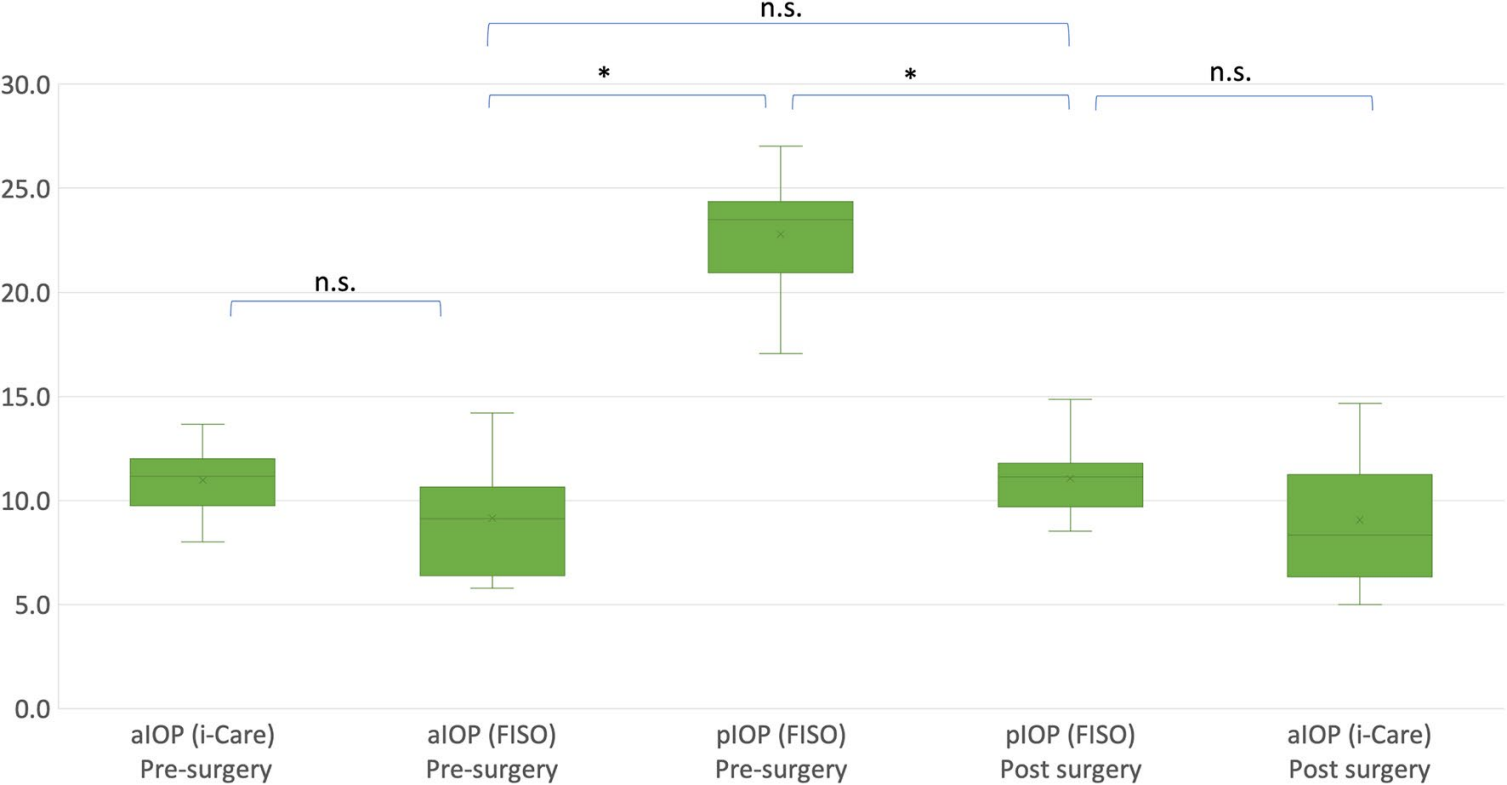
The box plot graphs showing the anterior chamber intraocular pressure (aIOP) and posterior vitreou-cavity pressure (pIOP) of the both eyes pre- and postvitrectomy of the six rabbits involved in the study. The intraocular pressure (IOP) was measured using an optic-fiber sensor (FISO) and conventional transcorneal rebound tonometry (i-Care). *P < 0.05, n.s.: not significant.

The measurement results for the aIOP and pIOP of the rabbits are shown in the graphs (Fig. 2). The mean aIOP using the i-Care and fiber-optic pressure sensor before vitrectomy were 11.0 (1.6) mmHg and 9.1 (2.6) mmHg, respectively, with no significant difference between the two types of measurement (*P* = 0.053). The mean pIOP previtrectomy was 22.8 (3.0) mmHg, which was significantly different from that of aIOP (P < 0.0001). After the vitrectomy, the aIOP measured using the i-Care was 9.1 (3.1) mmHg, which was not significantly different from the aIOP measured using the i-Care previtrectomy (*P* = 0.06). However, pIOP significantly decreased to 11.0 (1.7) mmHg compared with that before surgery (P < 0.0001), which decreased to nearly the aIOP (*P* = 0.043). The aIOP measured with the i-Care pre- and postvitrectomy (*P* = 0.42 and 0.63, respectively), the aIOP measured with the optic-fiber sensor (*P* = 0.68), and the pIOP pre- and postvitrectomy (*P* = 0.81 and 0.81, respectively) were not significantly different between the right and left eyes. Throughout the experiment, the rabbits were healthy without any complications such as intraocular infections.

## Discussion

This study confirmed that conventional rebound tonometry accurately detects the aqueous humor pressure, that the micro-optic sensor can measure both in aqueous and vitreous humor pressure accurately, and that the vitreous humor pressure is more than twice as high as the aqueous humor pressure under physiological conditions with intact lens and anterior vitreous tissue. The pressure difference between the aqueous and vitreous humor is resolved after vitrectomy surgery.

Measuring the vitreous humor pressure directly and accurately in vivo has been difficult so far; therefore, the pIOP remains unclear. Nagae et al. planned an ex vivo animal study using enucleated porcine eyes and reported that the pIOP, measured using the disc-shaped sensor originally developed, was almost the same as aIOP^25^. The sensor, however, had a width of 2 cm, thus necessitating a big incision for its installation into the posterior vitreous-cavity; this process could have destroyed the fragile vitreous structures and affected the result of the measurement. Equally, two ex vivo experimental studies to clarify pIOP using more fine instruments proposed that there might be a pressure difference between the two chambers^14,15^. Nevertheless, the authors concluded that a reliable measurement of pIOP could not be obtained because of the viscosity of the vitreous humor. Thus, we try to reveal the vitreous body pressure using the micro-optic-fiber sensor. Our preliminary experiment proved that the sensor provides reliable measurement even in vitreous bodies. The vitreous body is a loose collagen matrix mixed with the viscous liquid so that the hydrostatic pressure due to the body pushing on the sensing membrane of the sensor can register higher pressure than actual pressure. The micro sensor we used has an ultra-fine tip with force transduction through the silicone membrane that can lower the hydrostatic effect to a minimum. In addition, we measured the pressure 200 times/sec to avoid the noise of the mechanical force caused by the movement of the sensor. Thus, the sensor used in this study gave considerably minimum effect for the pressure measurement of the vitreous humor. Consequently, the main study has identified the pIOP within the vitreous humor in vivo for the first time and has demonstrated the quantitative difference between the two chambers. We need to note that previous ex vivo studies regarding pIOP ignore the effects of the adnexal structures such as orbital and eyelid pressure, which may be related to IOP. Specifically, we have proven that pIOP is significantly higher than aIOP. This finding is in good agreement with that of a previous study using a computer model^9,11,13^.

The observed difference between aIOP and pIOP in our study suggests that the vitreous body has higher pressure independently from the aqueous humor. The deviation can be produced because the vitreous humor is rigid and composed of a collagen type II fiber network with hyaluronic acid, thus giving it a gelatinous consistency^26,27^. Hence, the admittedly higher pIOP than aIOP may force the aqueous humor out from the posterior to the anterior cavity to produce the aqueous humor current^2,9,11,13^. The previous reports using the fluid dynamics model regarding the pressure difference of the two chambers have demonstrated the discrepancy in the outflow facilities. Specifically, the total pressure-dependent outflow of aqueous humor for the whole eye is more than twice as large as estimates based on Goldman’s equation, when focusing only on the aqueous humor^11,13^. The discrepancy corresponds to the pressure difference between the aqueous and vitreous humor shown in the current study; thus, the pressure difference might contribute to the outflow facility of the aqueous humor.

This study also revealed that the difference between aIOP and pIOP is resolved after core vitrectomy with the disruption of the anterior vitreous membrane. Vitreous fiber networks increase at the edge of the vitreous to form the bounding anterior and posterior hyaloid membranes^28^. Kawasaki et al. reported that the disruption of the membrane barrier increases the aqueous humor flow between the anterior and posterior cavity^29^. The current study showed that there was little pressure difference between the two cavity after the surgical removal of the anterior and core vitreous, thus corroborating the previous findings.

The current study gives us further interest in understanding the physical eye structure and the functional role of independent vitreous pressure, especially in glaucoma. In this study, the pIOP of the vitreous humor was 22.8 (3.0) mmHg, which corresponds approximately to the maximum limit of the normal aIOP of rabbits (23 mmHg)^30^. Thus, we should consider not only aIOP but pIOP in the physiological consideration of glaucoma. In addition, the rigid vitreous humor may cause shear stress on the neurofibril of the fundus, which is proportional to pIOP^9,31^. We cannot ignore the influence of the stress consideration of the unknown profile in retinal and optic nerve disease pathophysiology.

The current study has some limitations. Notably, we found no significant differences between aIOP measurements obtained using an optic-fiber sensor and rebound tonometry. Thus, we chose to exclusively employ one of the two methods for aIOP measurement in the subsequent experiment, primarily to minimize potential trauma to the animals. Similarly, we refrained from using both measurement methods for aIOP before assessing pIOP one week after the initial aIOP measurement. It is accepted that hypotony following ocular chamber cannulation typically resolves within a day^24^. Therefore, we assumed that aIOP would have recovered within a week following the initial measurement. In addition, we assumed that the order in which aIOP and pIOP were measured would not affect the results.

In conclusion, we focused on vitreous humor pressure and proposed that it should be considered separate from aqueous humor pressure, which may elucidate the discrepancy in previous studies regarding eye pathophysiology. The vitreous humors of rabbit eyes is a good intravitreal pharmacokinetic model of human eyes. Therefore, the result of this study may illustrate a good correlation with human eyes^32,33^. Further investigations of pIOP in human eyes are needed to evaluate the relationship between pIOP and aIOP in normal individuals and patients with various types of eye disease, which may help elucidate glaucoma pathophysiology and its progress in human eyes. Accordingly, pIOP may be linear with aIOP after core vitrectomy, which could be a key to resolving the longtime theme of coping with uncontrolled sight-threatening disease.

### Supplementary Information

Supplementary Information.

## Data Availability

We submit a supplemental file including all data.
